# Psychological and Gastrointestinal Symptoms of Patients with Irritable Bowel Syndrome Undergoing a Low-FODMAP Diet: The Role of the Intestinal Barrier

**DOI:** 10.3390/nu13072469

**Published:** 2021-07-19

**Authors:** Laura Prospero, Giuseppe Riezzo, Michele Linsalata, Antonella Orlando, Benedetta D’Attoma, Francesco Russo

**Affiliations:** Laboratory of Nutritional Pathophysiology, National Institute of Gastroenterology “S. de Bellis” Research Hospital, Castellana Grotte, 70013 Bari, Italy; laura.prospero@irccsdebellis.it (L.P.); giuseppe.riezzo@irccsdebellis.it (G.R.); michele.linsalata@irccsdebellis.it (M.L.); antonella.orlando@irccsdebellis.it (A.O.); benedetta.dattoma@irccsdebellis.it (B.D.)

**Keywords:** intestinal barrier, irritable bowel syndrome, low FODMAP diet, gastrointestinal symptom profile, psychological profile

## Abstract

A diet low in fermentable oligosaccharides, disaccharides, monosaccharides, and polyols (LFD) improves both gastrointestinal (GI) symptoms and the psychological profile of patients with irritable bowel syndrome with diarrhea (IBS-D). The effects of 12 weeks of LFD on GI symptom and psychological profiles in relation to inflammation and the involvement of the intestinal barrier were studied in twenty IBS-D patients. The IBS Severity Scoring System, the Symptom Checklist-90-Revised, the Italian version of the 36-Item Short-Form Health Survey, the IBS-Quality of Life (QoL) questionnaire, and the Psychophysiological questionnaire were administered. The GI barrier function was assessed by sugar absorption test, the serum and fecal zonulin levels, and the serum levels of intestinal fatty-acid binding protein and diamine oxidase. Interleukins (ILs) and lipopolysaccharide (LPS) serum levels were evaluated along with dysbiosis. At the end of LFD, GI symptoms, psychological state (mainly anxiety, somatization, psychoticism, and interpersonal sensitivity), and QoL significantly improved in these patients. Simultaneously, an improvement in small intestinal permeability and intestinal mucosal integrity occurred, while IL-6, Il-10, LPS, and fermentative dysbiosis significantly decreased. The LFD can modify the immune-inflammatory features and enhance intestinal permeability and mucosal integrity, thus determining a concurrent improvement in the clinical and psychological conditions.

## 1. Introduction

Irritable bowel syndrome (IBS) is a multifaceted disease in which the functional aspect is no more dominant for its etiopathogenesis [1]. Apart from the classical gastrointestinal (GI) symptoms recognized by the Rome criteria [2], several extra-GI symptoms have been described, including depression and anxiety [3].

Psychological factors are critical for the onset and persistence of IBS, and a strong correlation with the symptoms’ severity and the response to treatments has already been reported [4,5]. According to available data in the literature, the prevalence of anxiety and depression in IBS patients is 31.4% and 37.1%, respectively [6].

IBS is now considered a biopsychosocial disorder resulting from dysregulation of the interaction between the GI tract and the central nervous system (CNS). This hypothesis relies on the bidirectional relations between body and mind that delineate IBS as both GI and psychological disease [7]. Such a condition worsens IBS symptoms and lowers the quality of life (QoL) of patients, making a more changeling frame for its complex clinical presentations [8]. Anxiety and depression associated with IBS are still not formally recognized as comorbidity manifestations. However, recent studies have begun to clarify the different pathophysiological alterations that may account for their onset and perpetuation in IBS patients. Abnormalities in GI motility and sensitivity [9,10], altered intestinal permeability [11], impaired immune and peptide functions [12], and dysbiosis [13] may all dysregulate the so-called “gut–brain axis” connecting CNS and the GI tract [14]. This bidirectional communication [15] has been proven to be affected by the minimal inflammation and impairment of the intestinal barrier [16,17,18].

IBS pathophysiology has recently been linked to the altered small intestinal permeability (s-IP) associated with persistent low-grade immune activation [19,20]. In particular, significantly higher serum concentrations of inflammatory molecules and markers of bacterial translocation (e.g., interleukin—IL-6 and lipopolysaccharides—LPS) have been found in IBS patients with diarrhea (IBS-D) with concomitant increased s-IP in comparison with patients with IBS-D and normal s-IP [21]. Similarly, higher levels of LPS, circulating biomarkers of intestinal barrier integrity and gut dysbiosis have also been found more frequently in patients with depression or anxiety than in healthy patients [22].

Most IBS patients attribute their condition to some foods, such as the fermentable oligosaccharides, disaccharides, monosaccharides, and polyols (FODMAPs) [23,24], and many papers have demonstrated a positive effect of the low FODMAP diet (LFD) on the IBS-D symptoms [25]. However, limited data are still available on the LFD effects on non-GI symptoms, i.e., the psychological symptoms and impaired QoL, often complained about by these patients [26].

Dietary treatments are considered valid options to improve IBS symptoms, to be preferred to long-term pharmacological interventions whenever it is possible. Thus, the responses induced by appropriate diets could provide helpful information to investigate better the pathophysiology of the psychosocial factors of IBS [27]. The present study aimed to demonstrate that a long-lasting LFD can affect not only the GI symptoms but also the psychological profile and QoL in IBS-D patients. The link between psychological health, GI permeability, and intestinal barrier integrity was also investigated.

Specifically, the GI symptoms were assessed by the IBS Severity Scoring System (IBS-SSS). Psychological characteristics and stress were assessed through the Symptom Checklist-90-Revised Edition (SCL-90-R) and the Psychophysiological Questionnaire (QPF/R). QoL was assessed by the Italian version of the 36-item Short-Form Health Survey (SF-36) and the IBS-Quality of Life Questionnaire (IBS-QoL). GI permeability was studied by dosing the urinary recovery of non-absorbable sugars with different sizes such as lactulose, mannitol, and sucrose. The integrity of the intestinal barrier was assessed by measuring serum and fecal zonulin levels, intestinal fatty acid-binding protein (I-FABP), and diamine oxidase (DAO) serum concentrations. Serum LPS and urinary indole and skatole were measured as markers of intestinal dysbiosis. Finally, pro-inflammatory IL-6, IL-8, and TNF-α and anti-inflammatory IL-10 were also evaluated.

## 2. Materials and Methods

### 2.1. Patient Recruitment

Patients with IBS-D according to Rome IV criteria [28] were enrolled from January 2018 to May 2020 from among the outpatients of the National Institute of Gastroenterology “S. de Bellis” Research Hospital, Castellana Grotte (Bari), Italy.

The study protocol and the intervention diet have previously been published in open-access articles [18,25], and the clinical trial was registered on http://www.clinicaltrials.gov (NCT03423069)—last accessed data: 17 February 2021. The study was approved by the local Scientific Committee and the Ethics Committee of IRCCS Istituto Tumori Giovanni Paolo II—Oncological Hospital, Bari, Italy, 274/C.E. 12.12.17.

Before and after 12 weeks of LFD, patients were evaluated for their GI symptom profile, along with nutritional, biochemical, and psychological characteristics.

GI symptoms were analyzed by using a validated questionnaire, the IBS-SSS [29,30]. The questionnaire includes five items measuring the frequency and intensity of abdominal pain, the severity of abdominal distension, dissatisfaction with bowel habits, and IBS’s interference with daily life. In addition, it measures the frequency of evacuation. The IBS-SSS score ranges from 0 to 500, and patients had to have had a total IBS-SSS score >125 to be recruited into the study.

Along with dietary habits, a trained nutritionist evaluated physiological conditions, physical activity, and lifestyle. Patients followed their usual diet and filled in their eating habits diary every day. Together with eating habits to estimate daily energy intake, the diary included records of bowel habits, stool characteristics categorized following the Bristol stool form chart [31], drugs, and physical activity for calculating energy expenditure.

The psychological and QoL questionnaires and the biochemical variables evaluated in this research are described in the following paragraphs.

### 2.2. Psychological Questionnaire

#### 2.2.1. Symptom Checklist-90-Revised (SCL-90-R)

SCL-90-R is a commonly applied tool for self-reporting symptoms in psychopathology [32]. SCL-90-R evaluates a wide range of psychopathological symptoms, with nine main symptom dimensions and three global indexes. We only measured the Global Severity Index (GSI), since it best represents the intensity of psychological distress that subjects currently perceive. The raw score is converted to a T score, and a score equal to or higher than 63 is considered a clinically significant symptom [33].

#### 2.2.2. Psychophysiological Questionnaire—QPF/R

The QPF/R is a questionnaire that is part of the Cognitive Behavioural Assessment-2.0 (CBA-2.0), a test battery that provides a general overview of the psychological problems in the individual and social domain. It consists of 10 schedules, of which the QPF is contained in the sixth one. The QPF/R evaluates stress and psychophysiological disorders [34].

### 2.3. Quality of Life Questionnaire

#### 2.3.1. 36-Item Short-Form Health Survey (SF-36)

The Italian version of the SF-36 is a short questionnaire that measures the patient’s health condition. The subscales and the global indexes are organized so that the higher the score, the better the health status. The first three subscales reflect physical health (physical activity, limitations of role-specific activities due to physical problems, physical pain); the intermediate subscales reflect health in general (general health, vitality); the last three measure aspects of psychological-emotional health (limitations in social activity, restrictions of role-specific activities caused by mental health or emotional problems). There is an additional unscaled single item on changes in respondents’ health over the past year. For each variable, scores were coded, summed, and transformed on a scale from 0 (worst possible health state) to 100 (best possible health state). It is also possible to calculate the values of two global indexes: the first is related to physical health, the second to mental health. These values derive from the eight scales and allow all the scales to be summarized in just two scores. This questionnaire is intended to investigate the QoL of all patients suffering from any diseases [35].

#### 2.3.2. IBS Quality of Life Questionnaire

The IBS-QoL only surveys the QoL of patients with IBS. The questionnaire includes 30 items and is divided into nine subscales: Total score, Dysphoria, Interference with Activity, Body Image, Health Worry, Food Avoidance, Social Reaction, Sexual Concerns, and Relationship. The raw score is converted to a scale score ranging from 0 to 100, where a higher score indicates a better quality of life [36].

### 2.4. Sugar Absorption Test

All participants in the study were evaluated for GI permeability through a sugar absorption test. After fasting overnight, the subjects drank a test solution with lactulose (10 g), mannitol (5 g), and sucrose (40 g) in a volume of 100 mL. The evaluation of the three sugars in urine was performed by chromatographic analysis as previously reported [18,25]. The percentage of the ingested sugars (%lactulose, %mannitol, and %sucrose) was evaluated. In clinical practice, the urinary lactulose/mannitol ratio is used as a reliable parameter to evaluate the small intestinal permeability (s-IP). A lactulose/mannitol ratio higher than 0.030 was indicative of altered s-IP [37].

### 2.5. Biochemical Analyses

Blood samples were obtained after 10 h fasting from all patients. The subjects were not receiving anti-secretory treatment (including PPIs) 2 weeks before the evaluation. EDTA tubes were centrifuged at 2000× *g* for 15 min, and blood samples were stored at −20 °C until the assay was carried out. All the evaluations were performed using commercially available sandwich enzyme-linked immunosorbent assay (ELISA) kits. Serum and fecal zonulin were evaluated by using kits from Immunodiagnostik AG (Bensheim, Germany). Values lower than 48 ng/mL were considered normal for serum levels. Additionally, values lower than 107 ng/mL were considered normal for zonulin in feces.

I-FABP and DAO serum concentrations were evaluated by kits purchased from Thermo Fisher Scientific (Waltham, MA, USA) and Cloud-Clone Corp. (Houston, TX, USA), respectively. IL-6, IL-8, IL-10, and TNF-α circulating levels were measured by kits from B.D. Biosciences (Milan, Italy). LPS was evaluated by a kit obtained from Cloud-Clone Corp. (Katy, TX, USA).

### 2.6. Indican and Skatole Evaluation

All patients collected urine samples in the morning. A standard colorimetric assay kit (indican assay kit, ABnova Corporation, Taipei, Taiwan) was used according to the manufacturer’s procedure. The detection and measurement of skatole in urine were carried out on a high-performance liquid chromatography (HPLC) system using a 3-methylindole kit (EurekaLab Division, Chiaravalle, AN, Italy), as described previously [25]. Urine indican and skatole values higher than 20 mg/L and 20 μg/L are considered as indicators of fermentation and putrefactive dysbiosis [38,39].

### 2.7. Statistical Procedures

Unless otherwise stated, all results are expressed as mean ± SD. Non-parametric tests were applied to avoid violating the normal distribution assumption. The Wilcoxon matched-pairs signed-rank test was used to detect the differences between the IBS-SSS questionnaire items and the psychological and biochemical parameters before and after LFD. The IBS-SSS difference before and after treatment was used as the dependent variable, and the DAO, Somatization (subscale of SCL-90-R), and IBS-QoL total score were used as independent variables for linear regression analysis in a stepwise regression procedure. The explanatory variance (adjusted *R*^2^) was determined for the regression analysis and tested with the *F* test. The *t* values and their significance level were assessed to test the hypothesis that the contribution of the input variable (regression coefficient) is significantly different from zero.

Statistical differences were set at *p* < 0.05. Statistics were performed using Sigma Stat 11.0 (Systat Software, Inc., San Jose, CA, USA) and GraphPad Prism 5 (GraphPad Software Inc., La Jolla, Ca, USA).

## 3. Results

### 3.1. Anthropometric Characteristics of the Patients

Twenty patients who suffered from IBS-D completed the study. The anthropometric characteristics of the patients before and after LFD intervention are summarized in Table 1. Significant reductions in weight, BMI, and abdominal and body circumferences were observed.

### 3.2. Symptom Profile

Table 2 reports the effect of LFD on the single items of the IBS-SSS score. All items improved after LFD. Additionally, stool frequency reduced after diet compared with baseline.

**Figure 1:**
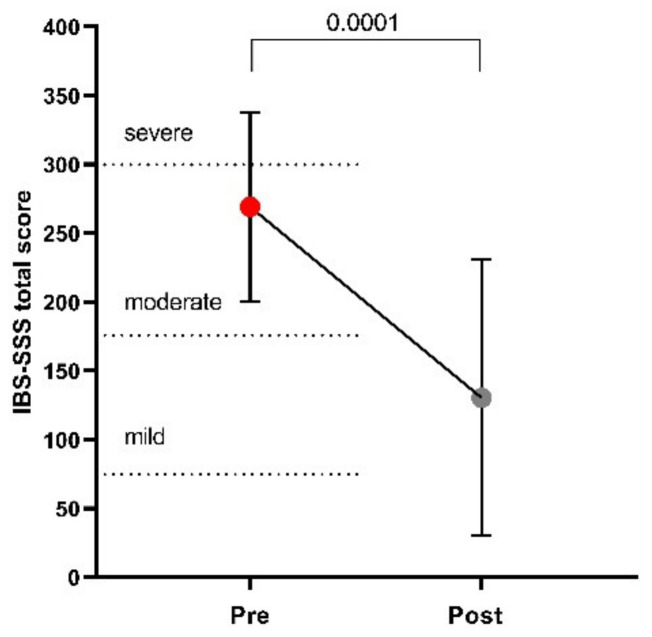
The IBS Severity Scoring System (IBS-SSS) total score before and after low FODMAPs diet. Data are presented as mean ± SD. Statistical analysis: Wilcoxon matched-pairs signed-rank test with a significant difference set at *p* < 0.05.

Figure 1 plots the total IBS-SSS score before and after LFD, showing a 52% reduction after diet, thus significantly decreasing the mean score values from moderate to mild (269 ± 69 vs. 130 ± 100; *p* = 0.0001).

### 3.3. Psychological and QoL Profiles

**Figure 2:**
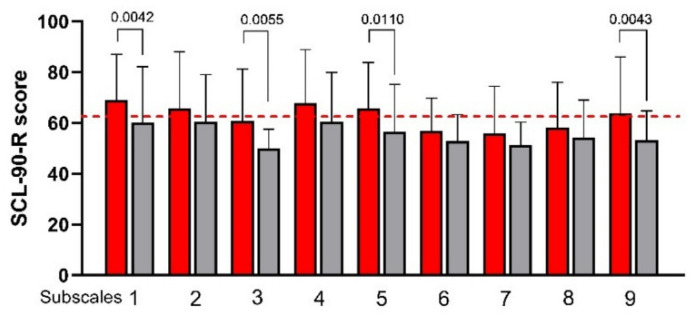
The Symptom Checklist-90-Revised (SCL-90-R) subscale scores before and after low FODMAPs diet. Data are presented as mean ± SD. Statistical analysis: Wilcoxon matched-pairs signed-rank test with a significant difference set at *p* < 0.05. Subscales: (1) Somatization; (2) Obsessive-Compulsive; (3) Interpersonal Sensitivity; (4) Depression; (5) Anxiety; (6) Hostility; (7) Phobic Anxiety; (8) Paranoid Ideation; (9) Psychoticism. The cutoff value is indicated by the red dashed line.

Figure 2 plots the subscales of the SCL-90-R score before and after LFD. Most of the SCL-90-R subscales were slightly above the cutoff value, specifically Somatization (1), Obsessive-Compulsive (2), Depression (4), Anxiety (5), and Psychoticism (9). After LFD, a significant decrease in SCL-90-R score was observed in four of nine subscales-namely, (1) Somatization, (3) Interpersonal Sensitivity, (5) Anxiety, and (9) Psychoticism. Depression reduced after LFD, showing only a trend toward significance (*p =* 0.0658). As expected, the LFD induced a significant reduction in the Global Index (GSI) score (69 ± 22 vs. 59 ± 19; *p =* 0.0034). Moreover, the measurement of stress and psychophysiological activation performed using the QPF/R showed a statistically significant reduction in its score after diet (69 ± 16 vs. 56 ± 13; *p* = 0.0002).

**Figure 3:**
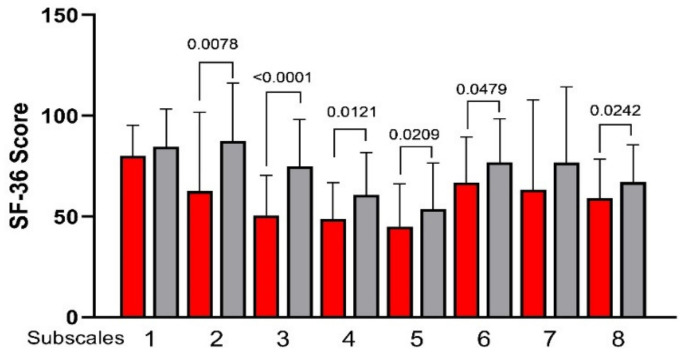
The 36-Item Short-Form Health Survey (SF-36) subscale scores before and after low FODMAPs diet. Data are presented as mean ± SD. Statistical analysis: Wilcoxon matched-pairs signed-rank test with a significant difference set at *p* < 0.05. Subscales: (1) Physical Function; (2) Role Physical; (3) Body Pain; (4) General Health; (5) Vitality; (6) Social Functioning; (7) Role Emotional; (8) Mental Health.

Figure 3 plots all the SF-36 subscales measured before and after the LFD. An evident and significant increase was observed in 6 of 8 subscales, specifically (2) Role Physical, (3) Body Pain, (4) General Health, (5) Vitality, (6) Social Functioning, and (8) Mental Health.

As for the two global indexes, the Physical Health scale significantly increased by approximately 15% after LFD (45.61 ± 6.14 vs. 52.62 ± 7.69, before and after diet, respectively; *p =* 0.0007) while the second scale, Mental Health, remained substantially unchanged (32.66 ± 18.21 vs. 38.06 ± 16.42; *p* = 0.1044).

**Figure 4:**
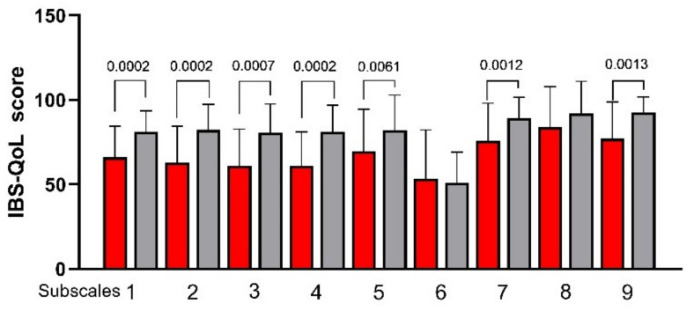
The IBS Quality of Life (IBS-QoL) total and subscale scores before and after low FODMAPs diet. Data are presented as mean ± SD. Statistical analysis: Wilcoxon matched-pairs signed-rank test with a significant difference set at *p* < 0.05. Subscales: (1) Total Score; (2) Dysphoria; (3) Interference with Activity; (4) Body Image; (5) Health Worry; (6) Food Avoidance; (7) Social Reaction; (8) Sexual Concerns; (9) Relationship.

Figure 4 plots the IBS-QoL subscales before and after LFD. (1) Total Score, (2) Dysphoria, (3) Interference, (4) Body Image, (5) Health Worry, (7) Social Reaction, and (9) Relationship significantly (*p* < 0.05) increased after LFD. Only the subscales (6) Food Avoidance and (8) Sexual Concerns were not affected by LFD.

### 3.4. The Small Intestinal Permeability (s-IP)

**Figure 5:**
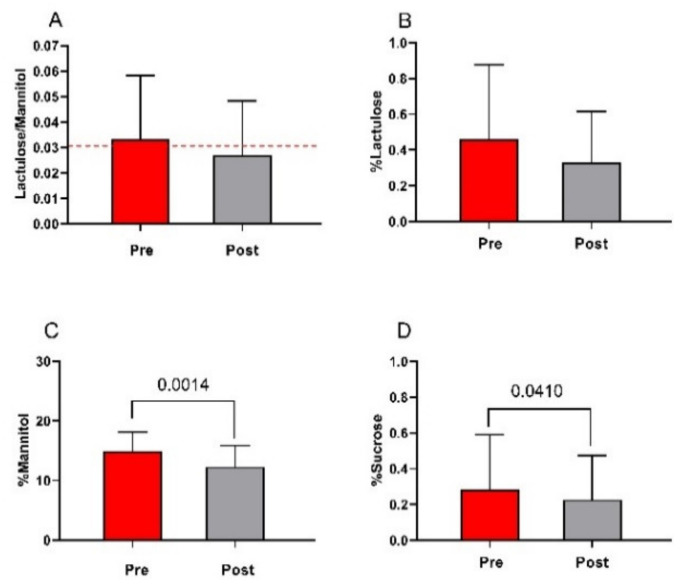
The small intestinal permeability (s-IP) before and after low FODMAPs diet. Panel (**A**) Lactulose to Mannitol ratio; panel (**B**) % lactulose; panel (**C**) % mannitol; panel (**D**) % sucrose. Data are presented as mean ± SD. Statistical analysis: Wilcoxon matched-pairs signed-rank test with a significant difference set at *p* < 0.05. The cutoff value is indicated by the red dashed line.

All the IBS-D patients underwent s-IP testing before and after LFD (Figure 5). At the beginning of the study, the lactulose/mannitol ratio was higher than the cutoff value of 0.030, indicating the presence of an altered s-IP. After the diet, the lactulose/mannitol ratio returned within the normal range of s-IP, although the 18% decrease did not reach statistical significance (0.033 ± 0.025 vs. 0.027 ± 0.021) (Figure 5, Panel A). As for the urinary recovery of lactulose (%lactulose), despite a 28% reduction, no significant modifications were found in IBS-D patients after the diet compared with baseline (0.46 ± 0.42 vs. 0.33 ± 0.29) (Figure 5, Panel B). Conversely, the %mannitol recovery in the urine significantly lowered by 20% at the end of treatment (15 ± 3.2 vs. 12.4 ± 3.6; *p =* 0.001) (Figure 5, Panel C). Lastly, %sucrose, a marker of gastroduodenal permeability, showed a significant 18% reduction after diet compared with the start of the study (0.28 ± 0.31 vs. 0.23 ± 0.25; *p =* 0.041) (Figure 5, Panel D).

### 3.5. Markers of Function and Integrity of the Intestinal Barrier

**Figure 6:**
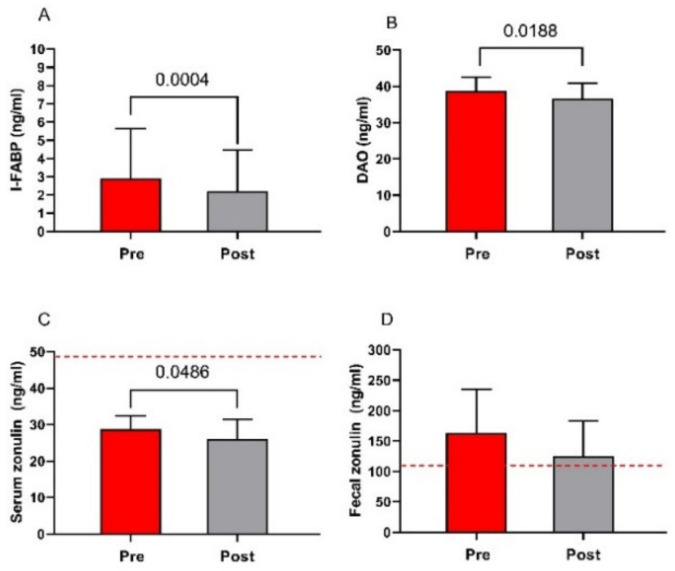
Biomarkers of intestinal barrier function and integrity before and after low FODMAPs diet. Panel (**A**) intestinal fatty acid-binding protein—I-FABP; panel (**B**) diamine oxidase—DAO; panel (**C**) serum zonulin; and panel (**D**) fecal zonulin. Data are presented as mean ± SD. Statistical analysis: Wilcoxon matched-pairs signed-rank test with a significant difference set at *p* < 0.05. The cutoff value is indicated by the red dashed line.

The markers of function and integrity of the intestinal barrier (serum levels of I-FABP, DAO, and zonulin along with fecal zonulin concentrations) in IBS-D patients are presented in Figure 6. The serum concentrations of I-FABP significantly lowered by 24% at the end of the diet (2.9 ± 2.7 ng/mL vs. 2.2 ± 1.3 ng/mL; *p* = 0.0004) (Figure 6, Panel A). Similarly, DAO levels significantly lowered at the end of the diet (39 ± 3.9 ng/mL vs. 37 ± 4.3 ng/mL; *p* = 0.0188) (Figure 6, Panel B). As for zonulin, serum concentrations significantly decreased at the end of the diet (29 ± 3.7 ng/mL vs. 26 ± 5.3 ng/mL; *p* = 0.0486) (Figure 6, Panel C). The basal fecal zonulin concentrations were far above the cutoff level of 107 ng/mL, and they lowered by 23% at the end of the diet, although not significantly and without falling within the normal range (163 ± 72 ng/mL vs. 126 ± 58 ng/mL) (Figure 6, Panel D).

### 3.6. Indices of Inflammation

**Figure 7:**
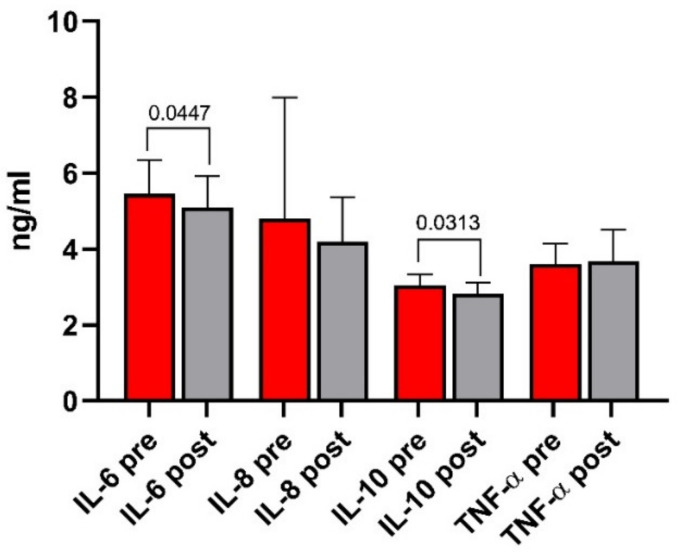
Circulating levels of IL-6, IL-8, IL-10, and TNF-α before and after low FODMAPs diet. Data are presented as mean ± SD. Statistical analysis: Wilcoxon matched-pairs signed-rank test with a significant difference set at *p* < 0.05.

The circulating levels of IL-6, IL-8, IL-10, and TNF-α in the IBS-D patients are presented in Figure 7. Only IL-6 (5.5 ± 0.9 ng/mL vs. 5.1 ± 0.8 ng/mL) and IL-10 (3.1 ± 0.3 ng/mL vs. 2.8 ± 0.3 ng/mL) levels significantly decreased at the end of the diet (*p =* 0.0447 and *p* = 0.0313, respectively).

### 3.7. The Markers of Bacterial Translocation and Intestinal Dysbiosis

**Figure 8:**
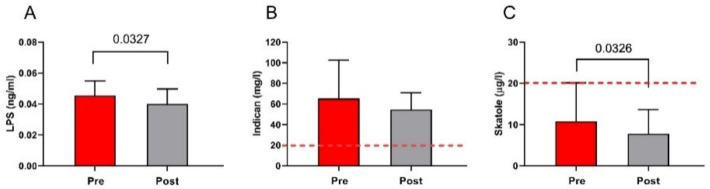
The markers of intestinal dysbiosis and bacterial translocation before and after low FODMAPs diet. Panel (**A**) LPS circualting concentrations; panel (**B**) urinary levels of Indican; panel (**C**) urinary levels of skatole. Data are presented as mean ± SD. Statistical analysis: Wilcoxon matched-pairs signed-rank test with a significant difference set at *p* < 0.05. The cutoff value is indicated by the red dashed line.

The LPS levels were significantly reduced by LFD (0.05 ± 0.01 ng/mL vs. 0.04 ± 0.01 ng/mL; *p* = 0.0327) (Figure 8, Panel A). As concerns the markers of dysbiosis, at the start of the treatment, the urinary indican concentrations were above the cutoff level of 20 mg/mL, indicating the presence of fermentative dysbiosis in the small intestine. However, the diet slightly reduced its concentrations, but without statistical significance (66 ± 37 mg/L vs. 55 ± 16 mg/L; *p* = 0.1281) (Figure 8, Panel B). On the contrary, at the beginning of the diet, the urinary skatole concentrations were within the limit of the normal range (below 20 µg/L). However, a significant decrease was observed at the end of treatment (11 ± 9.3 µg/L vs. 7.8 ± 5.8 µg/L; *p* = 0.0326) (Figure 8, Panel C).

### 3.8. Regression Analysis

Lastly, the regression analysis clearly shows that the variation of the IBS-SSS total score could be significantly described by a linear combination of three variables (namely, DAO, the SCL-90-R subscale Somatization, and the IBS-QoL Total score) (F = 14.95; df = 3; *p* < 0.001; adjusted *R*_2_ = 0.688) (Table 3). Therefore, IBS symptoms could be regarded as the sum of biochemical, psychological, and QoL parameters.

## 4. Discussion

The present study found that LFD improved GI symptoms, psychological state (mainly anxiety, somatization, psychoticism, and interpersonal sensitivity), and QoL of patients with IBS-D. Additionally, a concurrent improvement in small intestinal permeability and intestinal mucosal integrity occurred. Lastly, ILs and LPS significantly decreased as well, as fermentative dysbiosis was reduced after LFD.

As expected and already reported [18,25], a 90-day LFD significantly and positively affected the total and single-item scores of the IBS-SSS questionnaire. Additionally, the weight, BMI, abdominal, and body circumferences were significantly reduced after the treatment due to a long-lasting diet that introduced a lipid intake typical of the Mediterranean diet, which affected the bodyweight of patients assuming it.

An intriguing hypothesis regarding IBS mechanisms centers on an improvement in the microbiota composition and related amino acids production. Among the factors affecting IBS, reduced glutamine availability could lead to a deterioration of the intestinal barrier function and integrity. A recent randomized clinical trial has demonstrated that glutamine supplementation in patients with post-infectious IBS and impaired intestinal permeability improved stool consistency and frequency and reduced GI symptoms (mainly the IBS severity score) by contemporaneously improving intestinal permeability [40]. Poor gut health can disrupt the balance of neurotransmitters via the gut–brain axis, resulting in changes in psychological profile.

Of note, the psychopathological profile also improved, as demonstrated by the SCL-90-R scores, and in accordance with data in the literature [26]. Most IBS patients complain of comorbid psychiatric disorders, of which anxiety and depression are the most common [4]. Although our IBS patients did not suffer from psychiatric disorders, most SCL-90-R subscales were slightly above the cutoff value before starting the diet. Somatization, Interpersonal Sensitivity, Anxiety, and Psychoticism scores significantly reduced after diet, while Depression scores showed only a trend toward significance. These modifications refer essentially to those parameters mostly involved in the onset of IBS symptoms, especially in its diarrhea variant [41]. 

Noteworthy, LFD also positively affected the QoL of patients. At the end of the diet, the SF-36 and IBS-QoL questionnaires showed a significant improvement.

Available information on the LFD effects on QoL is still unclear and somewhat conflicting since some papers reported an improvement, while others failed to do so. These discrepancies are likely related to the different duration of the treatment and the type of IBS suffered from recruited individuals (i.e., diarrhea, constipation, or mixed variant) [42]. Conversely, when considering only IBS-D patients, our positive data aligned with those in the literature. The reduction of the osmotic action in the intestinal lumen due to FODMAPs and the correction of the “minimal inflammation” in these patients have both been considered possible determinants of the positive effects of LFD [43].

In this context, chronic inflammation and imbalance in inflammatory cytokines are involved in functional GI disorders such as IBS [44] and affect the neuroplasticity of the CNS, responsible, in turn, for the onset of psychological disorders [45]. In our study, IL-6 and IL-10 levels significantly decreased at the end of the diet. These results may contrast with the putative role of these ILs in IBS [46]. IL-6 is considered a pro-inflammatory cytokine, while IL-10 is known for its anti-inflammatory properties. However, a recent experimental model on stress-induced colonic inflammation demonstrated an increase in TNF-α and IL-10 and a concomitant decrease in tight junction proteins [47]. Therefore, it is conceivable that IL-10 could also exert a similar effect in IBS and that diet could reduce its levels and improve the mucosal barrier.

Beyond the intestinal inflammation, the pathophysiology of IBS-D seems to depend also on other factors, such as impairment of the intestinal barrier and the associated dysbiosis.

As demonstrated in our previous paper [25], the urinary percentage of mannitol decreased significantly at the end of the diet. The recovery of this non-absorbable sugar gives information on the transcellular pathway, and its reduction could be attributed to an improvement in food intolerance/malabsorption in the small intestine due to the reduced intake of FODMAPs. It is worth noting that the intestinal mucosal integrity markers (e.g., the serum levels of I-FABP, DAO, and zonulin) and LPS levels significantly decreased after LFD, thus confirming the positive action of LFD on the health of the intestinal barrier and the concomitant bacterial translocation [48,49]. The bacterial proliferation of the small intestine has been extensively demonstrated in IBS-D patients, and it is considered one of the potential culprits for the onset of IBS symptoms [50]. In our study, dysbiosis was indirectly investigated by assaying the urinary concentrations of indole and skatole [38, 39]. Beyond the statistical reduction after diet, indole levels in urine remained far above the cutoff level, indicating a persistent fermentative dysbiosis, which diet alone was not sufficient to treat. On the other hand, skatole was largely below the threshold value, although it significantly reduced after LFD without any clinical implication.

Interestingly, all the above-described modifications have also been associated with anxiety and depression [51,52]. Stevens et al. [22] reported a correlation between markers of increased intestinal permeability (namely, zonulin and I-FABP), altered stool fecal microbiota, and increased LPS levels in patients complaining of these psychological disorders.

Multiple regression analysis showed that the GI symptoms, expressed as the difference in IBS-SSS total scores between baseline and after LFD, could be significantly described by a linear arrangement of variables measuring mucosal barrier integrity (DAO), psychological processes (Somatization from SCL-90-R), and QoL (Total Score from IBS-QoL). In particular, the inclusion of the item Somatization among the predictors confirms the close relationship between abdominal pain and this psychological state, recently described by other authors [53]. Overall, our results, together with those available in the literature, suggest the gut as a new target for managing psychological distress, particularly in IBS patients, and that LFD can improve their psychopathological profile.

The amelioration in the mental health of these patients (mainly anxiety, somatization, psychoticism, and interpersonal sensitivity) demonstrates once more the close link between the GI tract and psychological functioning.

A unifying hypothesis linking CNS and functional GI disorders may be found in the latest reports about the involvement of the microbiota in microglia maturation and activation. According to this proposal, the microbial population in the gut can elicit psychological disorders (i.e., anxiety and depression) by causing a persistent inflammatory status and by affecting microglial functions [54].

The study has some weaknesses. Firstly, the research was not based on a double-blind design. It is known that this study design can be challenging to implement in dietary studies, as it cannot hide the characteristics of the diet from participants even when the researchers themselves provide it. Furthermore, a double-blind design makes it difficult to transpose the results obtained in real-life contexts and to personalize the diet in a long-term dietary condition. Secondly, in order to understand the complex IBS pathophysiological background, this study introduced a simplified model focusing on the changes in the intestinal barrier after LFD. Of course, several other factors could not have been considered. The selection of a homogeneous study group (i.e., stratified by sex, age, and BMI), a complete inflammatory profile in both serum and colonic biopsies, the role of intestinal peptides or amino acids need to be taken into account. Last but not least, dysbiosis was investigated by using an indirect analysis instead of more effective methods such as the evaluation of the sequences of bacterial 16S rRNA gene or its components. This method could have provided more information on the positive effects of this diet on IBS-D patients.

## 5. Conclusions

The data here shown confirm the simultaneous presence of GI symptoms and psychopathological alterations in IBS-D patients. Additionally, the “dual etiology” seems to be the best fitting model to explain the multifaceted profiles of IBS-D patients. The gut–intestinal microbiota–CNS axis can represent the unifying element in the chain of modifications of the gut barrier integrity and intestinal permeability to generate local and systemic effects. Therefore, an optimal treatment could be represented by a dietary approach that modifies the immune-inflammatory features and enhances the intestinal permeability, thus determining a concurrent improvement in the clinical and psychological conditions. The LFD seems to meet these conditions, and further studies will be needed to confirm this hypothesis.

## Figures and Tables

**Table 1:** Anthropometric data of the IBS-D patients before and after low FODMAPs diet.

	Pre	Post	*p*
Age (years)	42.65 ± 9.66	//	
Sex (M/F)	4/16	//	
Height (m)	1.63 ± 0.10	//	
Weight (kg)	65.16 ± 13.22	60.99 ± 12.22	<0.0001
BMI (kg/m^2^)	24.30 ± 4.16	22.84 ± 4.02	<0.0001
Mid-upper arm circumference (cm)	28.21 ± 3.67	26.68 ± 3.48	<0.0001
Shoulder circumference (cm)	109.90 ± 9.55	101.00 ± 8.49	<0.0001
Abdominal circumference (cm)	86.78 ± 10.25	83.79 ± 10.08	<0.0001
Hip circumference (cm)	77.78 ± 11.60	74.70 ± 10.04	0.0001
Waist circumference (cm)	97.18 ± 9.39	93.27 ± 8.53	<0.0001

BMI: body mass index. Data are presented as mean ± SD. Statistical analysis: Wilcoxon matched-pairs signed-rank test with a significant difference set at *p* < 0.05.

**Table 2:** The single items and stool frequency of IBS Severity Scoring System of IBS-D patients recorded before and after low FODMAPs diet.

	Pre	Post	*p*
Abdominal pain intensity	47.60 ± 22.15	20.25 ± 21.18	0.0003
Abdominal pain frequency	47.00 ± 28.12	20.00 ± 25.75	0.0001
Abdominal distension	51.63 ± 23.60	22.90 ± 20.29	0.0007
Dissatisfaction of bowel habit	61.45 ± 23.16	30.45 ± 22.92	0.0032
Interference with life in general	61.45 ± 18.43	36.80 ± 29.03	0.0054
			
Stool frequency (Bowel movements per day)	1.96 ± 0.78	1.18 ± 0.60	0.0012
Stool frequency (last 10 days)	1.79 ± 0.59	1.20 ± 0.61	0.0032

Data are presented as mean ± SD. Statistical analysis: Wilcoxon matched-pairs signed-rank test with a significant difference set at *p* < 0.05.

**Table 3:** Regression analysis between IBS-SSS total score and biochemical, psychological, and QoL parameters.

	β	Std. Error (β)	*p*	95% CI
DAO	10.58	4.42	0.029	2.88–18.27
Somatization (SCL-90)	4.65	1.41	0.005	2.11–7.20
Total score (IBS-QoL)	−3.13	1.12	0.013	−4.95–−1.31

All the variables were calculated as differences before and after the low FODMAPs diet. In the linear regression, the IBS-SSS was considered the dependent variable, the other factors being the independent variables. DAO = diamine oxidase.

## Data Availability

The datasets used and/or analyzed during the current study are available from the corresponding author on reasonable request.
